# Capturing Public Opinion on Public Health Topics: A Comparison of Experiences from a Systematic Review, Focus Group Study, and Analysis of Online, User-Generated Content

**DOI:** 10.3389/fpubh.2015.00200

**Published:** 2015-08-24

**Authors:** Emma Louise Giles, Jean M. Adams

**Affiliations:** ^1^Health and Social Care Institute, Teesside University, Middlesbrough, UK; ^2^MRC Epidemiology Unit, UKCRC Centre for Diet and Activity Research (CEDAR), Institute of Metabolic Science, University of Cambridge School of Clinical Medicine, Cambridge, UK

**Keywords:** incentives, health behavior, research methods, attitudes, thematic analysis, qualitative, quantitative

## Abstract

**Background:**

Capturing public opinion toward public health topics is important to ensure that services, policy, and research are aligned with the beliefs and priorities of the general public. A number of approaches can be used to capture public opinion.

**Methods:**

We are conducting a program of work on the effectiveness and acceptability of health promoting financial incentive interventions. We have captured public opinion on financial incentive interventions using three methods: a systematic review, focus group study, and analysis of online user-generated comments to news media reports. In this short editorial-style piece, we compare and contrast our experiences with these three methods.

**Results:**

Each of these methods had their advantages and disadvantages. Advantages include tailoring of the research question for systematic reviews, probing of answers during focus groups, and the ability to aggregate a large data set using online user-generated content. However, disadvantages include needing to update systematic reviews, participants conforming to a dominant perspective in focus groups, and being unable to collect respondent characteristics during analysis of user-generated online content. That said, analysis of user-generated online content offers additional time and resource advantages, and we found it elicited similar findings to those obtained via more traditional methods, such as systematic reviews and focus groups.

**Conclusion:**

A number of methods for capturing public opinions on public health topics are available. Public health researchers, policy makers, and practitioners should choose methods appropriate to their aims. Analysis of user-generated online content, especially in the context of news media reports, may be a quicker and cheaper alternative to more traditional methods, without compromising on the breadth of opinions captured.

## Background

Capturing public opinion on public health topics is important to gauge buy-in to political agendas (1), and to ensure that services meet the requirements of the public (2). By gauging public opinion, it is possible to determine wider community issues that may not have been identified if researchers or policy makers are the only constituencies involved in priority setting, intervention design, and delivery (3). Through understanding public opinion, research, policy, and practice can contribute to an informed evidence base, accounting for multiple stakeholder views (3). It has also been argued that the public have a moral right to be involved in publicly funded health research, which they have helped fund and which may have an impact on health care and public health interventions that they may receive (4, 5).

We are conducting a program of research on the effectiveness and acceptability of health promoting financial incentives (HPFIs) (6–9). These are cash, or cash-like, rewards or penalties provided contingent on behavior change, or non-change. Although a range of work, including ours, has confirmed HPFIs are effective in a range of contexts (9), less evidence is available on the acceptability of these interventions, including to the public (9, 10).

A number of methodological options are available for capturing public opinions on public health topics, such as HPFI. We conducted a systematic review, focus group study, and an analysis of user-generated online content. Here, we compare and contrast our experiences with, and results from, the three approaches, to help inform those researching public health issues about how each of the methods can help capture public opinion. All three studies have been reported in detail elsewhere (9–11). Our intention here is not to describe our methods and results in detail, but to compare and contrast the pros and cons of the three different methods.

## Methods

### Determining public opinion toward health promoting financial incentives using three different methods

**Figure 1:**
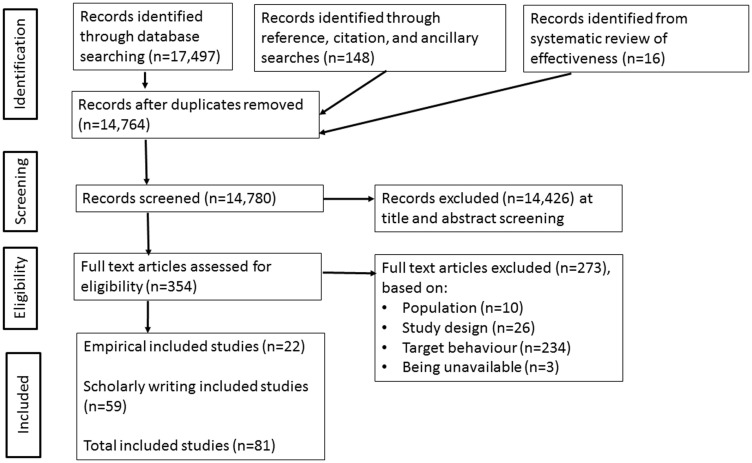
**PRISMA Flow Diagram**.

We conducted a systematic review of acceptability of HPFI (10). Unusually, we included both empirical and opinion pieces in this review as we believed both provided useful information on the acceptability of HPFI from a range of viewpoints (See Figure 1, PRISMA Flow Diagram). We searched a range of databases and the final review included 81 papers in total: 22 empirical studies and 59 opinion pieces. Methods and results are summarized in Table 1.

**Table 1:** **Methods and results of three methodological approaches**.

	Focus groups	Systematic review	Online user-generated content
**METHODS**			
Main approach	Eight focus groups (*n* = 74)	Searching of databases from the earliest date to October 2014	Analysis of 3,373 reader comments posted online in response to a news article on incentives
	*Inclusion criteria*: UK adults, aged 18+ years	*Databases searched*: Medline; Embase; Web of Knowledge; CINAHL; PsycINFO; ASSIA; Sociological Abstracts; Scopus; The Philosopher’s Index; Cochrane Library; SSCI; IBSS	*News article topic*: feasibility study of financial incentives for breastfeeding
	*Method*: face-to-face focus groups; audio-recorded; lasting on average 60 min	*Inclusion criteria*: English language title Published in a peer-reviewed journal Explored acceptability of financial incentives for healthy behaviors Acceptability explored in the public, policy makers, potential recipients, and practitioners	*Online news sites searched*: BBC; Guardian; Daily Mail; Telegraph; Independent; The Sun
	*Analytical approach*: thematic analysis using NVivo 10	*Analytical approach*: thematic analysis using NVivo 10	*Inclusion criteria*: popular websites defined as those that achieved an average monthly audience of at least five million unique viewers per month across laptop computers, desktop computers, and mobile devices in April and May 2013 *Analytical approach*: thematic analysis using NVivo 10
**RESULTS**			
Main themes	The nature of fair exchange	Fair exchange	Children are a lifestyle choice
	Design and delivery of incentive schemes	Design and delivery	Financial incentives for breastfeeding are discriminatory and divisive
	Effectiveness and cost-effectiveness	Effectiveness and cost-effectiveness	Creating a culture of entitlement
	Recipients	Recipients	Financial incentives for breastfeeding are potentially insulting
	Impact on individuals and wider society	Impact on individuals and wider society	Psychological impacts on recipients
	“Other” issues		Effectiveness and cost-effectiveness
			Generating initial motivation
			Design and delivery
			Informed choice

Our focus group study included 74 participants in eight groups, stratified by age and socio-economic group. These explored participant views on financial incentives for a range of different health behaviors. Data were analyzed using thematic analysis and results were summarized in Table 1.

Finally, we conducted a thematic analysis of online user-generated content (12). This was inspired by “netnography” (13–15), a technique originally established in the marketing domain to capture consumer opinions toward products and services (13–15). The approach has been relatively underused in the public health arena (16, 17), although there is growing recognition of the value that online media can have for exploring public health issues (17–21). This was an opportunistic piece of work arising from extensive UK coverage of a pilot study offering financial incentives for breastfeeding to mothers living in a deprived area (22). Online news coverage of the pilot trial generated substantial reader comments in response. In total, 10 articles were identified from popular UK news websites, with over 3,000 reader comments posted. We uploaded these reader comments into NVivo software for thematic analysis (23). Results are summarized in Table 1.

Across all three methods, many of the main themes are comparable, such that financial incentives – if they are to be accepted – need to be fair to all individuals, be effective and cost-effective, and be carefully designed so as not to increase inequalities or have a negative impact on recipients. The analysis of the online user-generated content revealed slightly more themes in terms of children being a lifestyle choice, and that it is important to understand the impact incentives have on intrinsic motivation for behaviors. This could be due to the fact that this analysis focused on one behavior only – breastfeeding – whereas the focus groups and systematic review covered a range of healthy behaviors (e.g., smoking cessation, physical activity and so on).

## Comparing Experiences of Three Methods for Capturing Public Opinion

Table 2 summarizes what we felt were the pros and cons of the three methods discussed.

**Table 2:** **A comparison of methodological approaches**.

Method	Advantages	Disadvantages
Systematic review	Allowed:	Required multiple skill sets – e.g., an information scientist and researcher (33)
	searching of an extensive range of data sources and the use of an exhaustive search strategy (24)	There was limited guidance available on how to critically appraise non-standard included papers (i.e., scholarly critique) (32, 34)
	tailoring of the research question (25) the aggregation of data (26–29)	May have excluded papers that were not indexed in the main databases (31, 35, 36)
	the identification of what is and is not known on the topic	Requires updating to remain current (37)
	us to answer a question on acceptability where quantitative data may have been insufficient (30–32)	Narrative reviews are sometimes viewed to hold less weight (36, 38)
Focus groups	Allowed:	Participants may have conformed to the majority view (41)
	consensus to be achieved and identification of outlying opinions (39)	Are costly in terms of human resources (42)
	probing of “new” issues	Results may not be generalizable (although could be transferable) (41, 43)
	adherence to best practice, given a wealth of guidance available (40)	
Thematic analysis of online content	Was a timely and inexpensive approach to data collection (17)	Ethical issues were raised, such as privacy concerns (44)
	Established a wide range of opinions using a large sample size Allowed for objectivity	Being removed from the commenting process meant that we could not probe individuals to elaborate on their comments
		The sample may not be representative of the general population (45) as user characteristics are often unavailable

We found the thematic analysis of user-generated online content to be a timely and inexpensive method that allowed us to explore a wide range of public opinions in response to incentives for breastfeeding (17, 46). It was a quick process in that comments were downloaded, “cleaned” and made ready for analysis in a few hours, with the full analytical process taking a matter of weeks. This is in comparison to the much longer timescale for searching and screening in the systematic review, and recruiting participants and arranging and conducting focus group interviews. The inclusion of more than 3,000 reader comments in the analysis of online content also provided a much larger sample size than could have been reasonably achieved using traditional qualitative methods, or even a quantitative survey in the same timescale (47). Whilst greater sample size is not necessarily good in its own right in qualitative research, this volume of data did give us much greater confidence that thematic saturation had been achieved than in the focus group study.

However, the analysis of user-generated online content was not without its limitations. Using comments posted to online discussions for research purposes raises a number of ethical considerations (44). As the comments were publically available, and because the websites state in their privacy policies that site content can be used in other ways, we deemed it ethical to use the comments, despite the lack of explicit informed consent from participants. We sought permission from the news sites to use these comments for research purposes and abided by copyright guidelines (48). We also gained ethical approval from Newcastle University and upheld ethical best practice throughout, including adhering to data protection, treating data confidentially and ensuring participant anonymity. We spent much more time considering the ethical implications of the analysis of online content than the focus group study partly because standard guidance for this type of work is not available. Whilst far from commonplace, using online data for research is no longer particularly novel. The research community needs to move faster in developing guidance and norms of ethical practice for these newer research contexts.

Unlike in the focus groups, we chose not to take part in, and steer online discussions, or identify ourselves as non-participating observers to others commenting on included articles. Instead, we decided, as others have done (49), to remain anonymous and to simply download posted comments. This ensured we did not influence the discussion process and provided an additional degree of objectivity to data collection, compared to the focus groups (44, 50). However, it also meant, unlike in the focus groups, that we were unable to probe respondents for clarification on what particular comments meant and their reasons for writing them (44, 46).

Whilst systematic reviews aim to capture all extant research meeting particular criteria and hence be “representative” of the available research, individuals who comment on websites or take part in focus groups may not be representative of the wider population (47). They may, for instance, be socially similar to each other, or hold particularly strong viewpoints on a given topic (45). However, both of these are limitations of any opinion-based research (either qualitative or quantitative) which participants must opt-in to. Furthermore, it is possible that individuals may be more likely to be truthful in the partially anonymous space of the internet, compared to in a face-to-face setting (51, 52). One advantage of research methods where individuals come in direct contact with researchers is that questions concerning demographics can be asked, meaning that conclusions concerning representativeness can be drawn. This was not the case with our analysis of online content.

In both the systematic review and online study, we had to sift through data that was not directly relevant to the research. In both contexts, a balance between sensitivity and specificity was needed, in identifying data relevant to our research aims whilst interpreting the comments in a way that was true to the meaning intended by the commenters (53, 54). Arguably the online study, which we were able to immediately focus by restricting inclusion to comments on articles of direct relevance to the research, achieved this balance more effectively.

A final consideration relates to debriefing participants. Arguably a systematic review publication is, itself, a summary of findings for “participating” authors. In focus group research, it is normal to explain the reason for the research to participants during recruitment, and summarize discussions at the end of group interviews for immediate feedback to participants. Participants may also be offered the opportunity of receiving a fuller summary of all results at a later date. We considered something similar in our online study – perhaps by posting a summary of our results on the websites from where comments were downloaded. This would allow readers to remark on the findings and for commenters to see our interpretation of their comments. We decided not to do this because we felt these would be likely to be seen by only a very small minority of those who originally contributed to the research. Others have identified that debriefing participants is more complicated in internet-based forum, compared to traditional research (44) and further consideration of whether and how this should be done is required.

Originally, we expected that the analysis of user-generated online content would generate a different set of opinions to those captured in our systematic review and focus groups. This is because the latter involved a different, perhaps more vocal, population discussing the particularly emotive topic of breastfeeding. However, in general, the results from our analysis of online reader comments reflected the findings of both the systematic review and the focus groups (see Table 1). Thus, we are confident that using online data and tools to determine public attitudes toward HPFI (and perhaps also other topics), accurately captures the range of public opinions extant.

Other researchers have reported similar findings. For example, Henrich and Holmes (2011) found, when they undertook a thematic analysis of online news reader comments in Canadian news media toward the H1N1 vaccine, similar opinions to those which had previously been established via focus groups and surveys (18). This suggests that thematic analysis of reader comments to online news coverage is an appropriate method for a variety of public health topics and in a variety of contexts (19, 43, 55). However, it is worth remembering that sampling and response bias will occur when using an online sample (45, 56). In order to limit this bias, we attempted to detect differences in comments according to the stance and publication site of difference articles, but did not find any particular patterns. These are biases that are hard to detect or eliminate, but, as discussed above, these problems are not unique to analysis of online user-generated content.

### Using analysis of user-generated online content more widely

Given the similarity of findings across the three methods used, and the relative novelty of the analysis of user-generated online content, we focus the remainder of our discussion on this method in particular. Despite the benefits we feel it offers, it is worth highlighting that analysis of user-generated online content would only be a viable methodological option if the topic of interest is covered online in a context allowing for user-generated response and discussion. In the example, we used reader responses to online news media reporting, and so the method is restricted to issues that are covered in online news stories and by outlets that allow for reader comments. This could include user-generated content available in other fora – although some, more closed, contexts (e.g., member-only discussion boards) may raise additional ethical issues. Where researchers wish to be proactive and gain public feedback on a particular issue, the use of a press release may help stimulate news coverage and hence reader comments. In our case, the topic of financial incentives for breastfeeding was well-covered by UK news sources.

Where a limited evidence base exists, analysis of user-generated online content could enable researchers to achieve a preliminary grounding in a topic prior to conducting further, more resource-intensive work. It has also been suggested that the number of comments that are posted in response to a news article indicates strength of feeling toward an issue (18).This would be potentially useful to researchers, but also to policymakers who may wish to gauge the strength of public opinion and reaction to a new policy or to a change in policy. Although, again, issues of sampling bias are important to remember. This approach could help identify areas of divergence between the views of the public and policymakers, helping policymakers to design policy which is likely to be more accepted by the public (18). Certainly it is argued that *“good public discourse is maximally polyvocal, and good public policy must incorporate, hence accommodate all agents, rather than representing a single interest”* (52).

Analysis of user-generated online content could also indicate to policymakers where further public health information or education is required. This was an important finding from our work where we identified, for example, a public perception of insufficient information concerning the benefits of breastfeeding.

Furthermore, where issues are particularly sensitive, the use of an internet-based (and anonymous) forum may help individuals to feel more comfortable about expressing their opinions. This could elicit more truthful opinions than would be gained in a face-to-face setting (52). It has also been argued that posting comments online can help to build a sense of community amongst those participating, helping individuals to feel more confident when posting their comments (20, 57). Additionally, even when posted comments are potentially offensive, shocking and left-field, they still enable researchers to capture a representation of the full range of a “social and cultural phenomena” (52). Such breadth of opinion may be less likely to be captured in face-to-face settings where social processes inhibiting unusual opinions may be more overt. Certainly, the media can play a large role in the public’s responses to health issues (21) and as such using responses to the media as a resource to garner public opinion seems expedient.

In terms of resources, if these are restricted, analysis of user-generated online content provides an opportunity for in-depth qualitative analysis using accepted and rigorous analytical methods at a fraction of the time and cost. Thus, such analysis of online news comments may be particularly useful to policymakers, with limited resources, exploring public attitudes to new and controversial interventions that have attracted media attention. Timely examples include minimum unit pricing of alcohol, e-cigarettes, and standardized cigarette packaging.

Finally, it is probably important that the online news sources from which reader comments are taken for analysis, are viewed to be credible. In these terms, credibility of news sources may be perceived very differently by different audiences. Credibility could be enhanced by balanced coverage and appropriate citation of evidence within news coverage. Exploring, as we did, whether there are obvious differences in user responses according to the content of coverage can also give more trustworthiness to conclusions (18). Should researchers and policymakers wish to extend this method beyond topics covered in news stories, to, say, public opinion on health advice, information or content on other websites, they would have to consider the trustworthiness, design and perceived credibility of those websites (19). These issues could all impact the number of people who would view the website, whether they view the information that the website contains to be reputable, and whether they take the time to comment (19).

## Conclusion

Whilst researchers should choose methodologies appropriate to their research questions, if a large sample, up-to-date public opinions (47), and a relatively robust data source is required, then analysis of online news content confers benefits similar, and even additional to, other methodological approaches. Further empirical work is required to confirm the validity of this method and representativeness in terms of the range of opinions garnered in a wider range of contexts (47). Greater debate on the ethical issues raised by this approach and standard guidance for research in this context is also warranted (58).

## Author Contributions

EG conceived of, and drafted, the manuscript. JA revised the manuscript. All authors read and approved the final manuscript.

## Conflict of Interest Statement

The authors declare that the research was conducted in the absence of any commercial or financial relationships that could be construed as a potential conflict of interest.
